# Dental Protective Association

**Published:** 1889-03

**Authors:** 


### DENTAL PROTECTIVE ASSOCIATION.

We call attention to the new circular of the Dental Protective Association on pages 46–47, in our advertising columns, and advise every reader of this issue to send for Constitution and By-Laws, or what is better, send Dr. J. N. Crouse a check for $10 at once, and thus protect yourselves against suit or injunction for using crowns or bridges. The association agrees to defend each and every individual member of the profession, out of the general fund which we are happy to say is rapidly increasing. Let the profession organize and protect itself.

